# Adam19 Deficiency Impacts Pulmonary Function: Human GWAS Follow-up in Mouse

**DOI:** 10.21203/rs.3.rs-4207678/v1

**Published:** 2024-04-08

**Authors:** Huiling Li, John House, Cody Nichols, Artiom Gruzdev, James Ward, Jian-Liang Li, Annah Wyss, Ezazul Haque, Matthew Edin, Susan Elmore, Beth Mahler, Laura Degraff, Min Shi, Darryl Zeldin, Stephanie London

**Affiliations:** National Institute of Environmental Health Sciences; National Institute of Environmental Health Sciences; Whitsell Innovations, Inc.; National Institute of Environmental Health Sciences; National Institute of Environmental Health Sciences; National Institute of Environmental Health Sciences; Beth Israel Deaconess Medical Center; National Institute of Environmental Health Sciences; National Institute of Environmental Health Sciences; National Institute of Environmental Health Sciences; National Institute of Environmental Health Sciences; National Institute of Environmental Health Sciences; National Institute of Environmental Health Sciences; National Institute of Environmental Health Sciences; National Institute of Environmental Health Sciences

**Keywords:** meltrin beta, RNA-Seq, lung function, flexiVent, spirometry, inflammation

## Abstract

**Purpose:**

Over 550 loci have been associated with human pulmonary function in genome-wide association studies (GWAS); however, the causal role of most remains uncertain. Single nucleotide polymorphisms in a disintegrin and metalloprotease domain 19 (*ADAM19*) are consistently related to pulmonary function in GWAS. Thus, we used a mouse model to investigate the causal link between *Adam19* and pulmonary function.

**Methods:**

We created an *Adam19* knockout (KO) mouse model and validated the gene targeting using RNA-Seq and RT-qPCR. Contrary to prior publications, the KO was not neonatal lethal. Thus, we phenotyped the *Adam19* KO.

**Results:**

KO mice had lower body weight and shorter tibial length than wild type (WT). Dual-energy X-ray Absorptiometry indicated lower soft weight, fat weight, and bone mineral content in KO mice. In lung function analyses using flexiVent, compared to WT, *Adam19* KO had decreased baseline respiratory system elastance, minute work of breathing, tissue damping, tissue elastance, and forced expiratory flow at 50% forced vital capacity but higher FEV_0.1_ and FVC. *Adam19* KO had attenuated tissue damping and tissue elastance in response to methacholine following LPS exposure. *Adam19* KO also exhibited attenuated neutrophil extravasation into the airway after LPS administration compared to WT. RNA-Seq analysis of KO and WT lungs identified several differentially expressed genes (*Cd300lg, Kpna2, and Pttg1*) implicated in lung biology and pathogenesis. Gene set enrichment analysis identified negative enrichment for TNF pathways.

**Conclusion:**

Our murine findings support a causal role of ADAM19, implicated in human GWAS, in regulating pulmonary function.

## Introduction

Spirometric measures of lung function are routinely used in clinical medicine to diagnose chronic obstructive pulmonary disease (COPD) and monitor its severity along with asthma and other lung diseases. Lower function is related to mortality independently of other risk factors [1]. Human genome-wide association studies (GWAS) have identified genetic variants in over 550 genes related to pulmonary function [2]. Among these, variants in a disintegrin and metalloproteinase domain 19 (*ADAM19*) have been consistently associated with forced expiratory volume in the first second (FEV_1_) [3, 4], the ratio of FEV_1_ to forced vital capacity (FVC) [1–3, 5–8], peak expiratory flow (PEF) [3, 4], and COPD [9, 10]. However, while GWAS is powerful for identifying genetic associations, it cannot assign causality. Therefore, we followed up on these human GWAS findings using mouse models.

ADAM19 protein is primarily membrane-bound in various tissues and is expressed in the lung [11, 12]. It functions by shedding proteins, such as tumor necrosis factor (TNF), from the cell membrane by activating the catalytic site in its exon 11 [13–15]. Shed proteins can trigger signal transduction and regulate inflammation and other pathological processes [16–18].

The original study of genetic disruption of *Adam19* in mice showed it to be essential for cardiac development [19]; mice deficient in *Adam19* exons 10–12 exhibited severe cardiac defects, with only 5% surviving to postnatal day 8. Therefore, expecting early lethality and lack of specific ADAM19 antibodies, we created a reporter mouse by replacing exons 6 and 7 in *Adam19* with a tdTomato red gene construct. We expected the heterozygous reporter mouse to be viable and thus usable to visualize the tissue distribution of ADAM19 and study the role of *Adam19* in organogenesis, especially of the lungs. Mice with homozygous *Adam19-tdTomato* alleles are equivalent to *Adam19* knockout (KO); surprisingly, they were viable. Thus, we performed RNA sequencing (RNA-Seq) and reverse transcription-quantitative polymerase chain reaction (RT-qPCR) to validate the knockout of *Adam19*. We confirmed the knockout and measured pulmonary function in adult *Adam19* KO mice and WT controls. We also measured body weight, tibial length, and body composition. Further, given the involvement of *Adam19* in inflammation, we examined whether genetic deficiency of *Adam19* affects airway responsiveness and the immune cell profile of bronchoalveolar lavage fluid (BALF) following LPS administration. Some of the results from these studies were previously presented as an abstract [20].

## Materials and Methods

Detailed methods are in the online supplementary information.

### Adam19 Gene Targeting Scheme and Murine Studies

**Figure 1:**
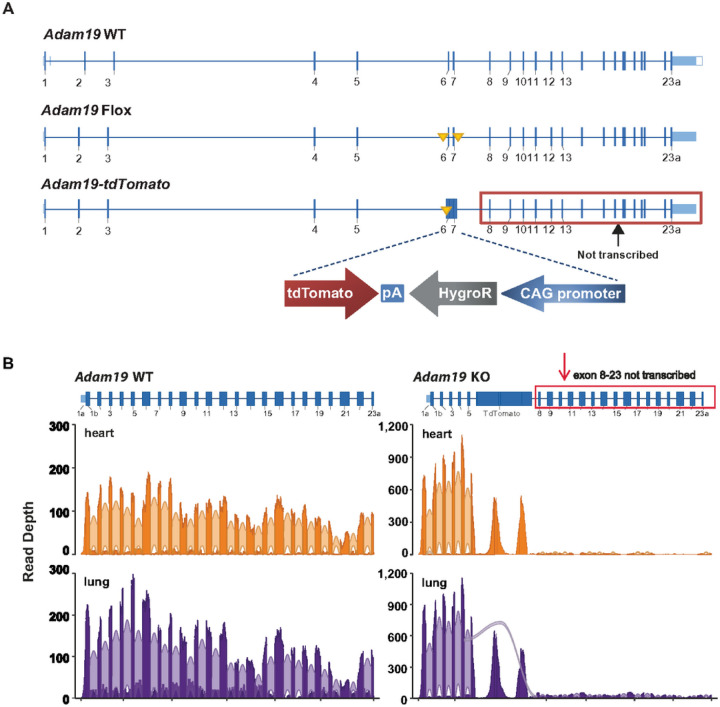
Gene targeting scheme (A) for Adam19-tdTomato allele and validation by RNA-Seq (B). A. Adam19 WT: Endogenous wild-type locus. Adam19 Flox: Adam19 conditional null (“flox”) allele with exon 6 and 7 floxed by LoxP sites (solid yellow triangles). Adam19-tdTomato: Adam19 mutant allele in which exons 6 and 7 are replaced by the tdTomato construct, disrupting Adam19 gene expression. Homozygosity for Adam19-tdTomato alleles is equivalent to Adam19 knockout (KO). Each blue box represents an exon; the exon number is underneath. pA: polyA; hygroR: Hygromycin Resistance. CAG: CMV enhancer, chicken beta-Actin promoter, and rabbit beta-Globin splice acceptor site. B. Read densities of Adam19 exons and junctions in WT and KO mice by RNA-Seq analysis. The blue boxes represent exons. In the Adam19 KO, exons 6 and 7 were replaced by the tdTomato construct and showed minimal transcript expression from exon 8 through the end of the Adam19 gene. Bold orange or bold purple regions represent aggregate sequence read depth across the Adam19 WT (left panel) or KO (right panel) gene locus. Light orange or light purple arcs indicate sequence reads whose alignments represent observed splice junctions. The arc width indicates the aggregate number of junction reads. No in-frame splicing events were detected from exon 5 to any downstream Adam19 exons. n=3 mice per genotype per heart or lung tissue.

*Adam19* exons 6 and 7 were replaced with an in-frame tdTomato construct. The homozygous *Adam19-tdTomato* allele is equivalent to the *Adam19* KO (Fig. 1A).

Mice in this study were all males aged 9–13 weeks with 129S6/SvEvTac background, confirmed by MiniMUGA genome genotyping arrays [21], performed by Transnetyx, Inc. (Cordova, TN). The use of animals followed NIH guidelines and was approved by the NIEHS Animal Care and Use Committee.

### RNA-Seq and RT-qPCR

Strand-specific RNA-Seq was conducted on Illumina NextSeq. Sequence coverage was visualized with sashimi plots [22]. The absence of *Adam19* transcript in KO was validated by both RNA-Seq and RT-qPCR.

### Differential Gene Expression and Gene Set Enrichment Analysis

RNA transcript reads were quantified versus GENCODE vM33 comprehensive transcripts (mm39) using Salmon 1.10.0 [23]. Differential gene expression was performed by limma-voom 3.54.2 [24], using thresholds for false discovery rate (FDR)-adjusted *p* < 0.05, fold change ≥ 1.5, and a group mean normalized transcript abundance ≥ 6 in at least one sample group.

We used the gene set enrichment analysis (GSEA) [25] to identify significantly enriched pathways (FDR *p* < 0.25) for genes differentially expressed between *Adam19* WT and KO by RNA-Seq datasets. The analysis was conducted using the Broad Molecular Signature Database (MSigDB, v2023.2.Mm) hallmark gene sets collection.

### Embryo Organogenesis

E18.5 embryos were collected, sectioned sagittally (5 μm thickness), and stained with hematoxylin and eosin. A pathologist (SAE) evaluated the tissue sections, focusing on the cellular structure of the heart, lung, valves (heart, aortic, and pulmonary), adrenal glands, and diaphragm.

### Assessment of Body Weight, Tibia Length, and Body Composition

Body weight was measured using a top-loading scale with an accuracy of 0.01 g. Tibia length was measured with a ruler with an accuracy of 0.1 cm. Body composition parameters [26] were assessed using the Faxitron Dual-energy X-ray Absorptiometry (DXA) imaging system.

### Pulmonary Function Parameter Measurements in Mice

Pulmonary function parameters were measured using flexiVent FX2 with a negative pressure-driven forced expiratory extension. Baseline measurements and responses to methacholine doses were assessed [27, 28]. For LPS experiments, mice received LPS or saline via oropharyngeal aspiration (OPA). After four hours, lung function parameters were measured using the same flexiVent procedure.

### Immune Cell Profile and Cytokine Analysis in BALF in LPS Exposed Mice

BALF was collected from each mouse by rinsing the airways [27]. We counted cells using an automated cell counter and determined the percentage of different immune cell types from cytospin slides. For LPS experiments, mice received LPS or saline via OPA and were euthanized after 4 hours. BALF was then collected and analyzed as described above. Cytokine concentrations (IL-1b, IL-2, IL-6, KC, MCP-1, MIP-1a, MIP-1b, and TNF-a) were determined using a custom Bio-Plex Pro Mouse Cytokine 8-plex assay.

### Statistical Analyses

We conducted linear regression analyses to assess genotype differences in body weight, body composition parameters, and tibia length. Genotype differences in lung function parameters were analyzed at baseline using a general linear model, and the maximum response to methacholine doses (normalized to response to PBS aerosol) was analyzed using a linear mixed-effect model with a random intercept. Differences in dose-response slope changes by LPS were assessed between genotypes. All lung function parameters were adjusted for body weight. Changes in cell counts of each immune cell type (except eosinophils present in just one mouse) across genotypes following LPS exposure were analyzed using linear regression. Linear regression with a robust sandwich estimator was used for cytokine data analysis. We used R version 4.2.2 to run statistical analyses and plots.

## Results

### Gene Targeting Scheme and Validation for *Adam19* KO Mouse

We replaced the *Adam19* exons 6 and 7 with the dTomato red gene open reading frame and anti-sense Hygromycin resistance (HygroR) cassette (Fig. 1A). RNA-Seq confirmed the absence of *Adam19* transcript encoding the active catalytic site of the functional protein. In both heart and lung, *Adam19* mRNA expression was minimal from exon 8 through 23 in KO mice (Fig. 1B and S1). Small amounts of exon 5 to exon 8 splicing transcripts were observed in the lung but not the heart in KO mice (Fig. 1B).

The detailed gene transcript structure in the heart and lung (WT and KO), derived from RNA-Seq analysis, is shown in Fig. S1 (available via the NIEHS-hosted track hub in the UCSC Genome Browser using the URL:https://bit.ly/adam19tdt). The RNA-Seq data discussed here were deposited in NCBI’s Gene Expression Omnibus [29] under the accession number GSE183318 (https://www.ncbi.nlm.nih.gov/geo/query/acc.cgi?acc=GSE183318, reviewer token: ubgnmiignvgnbix).

Follow-up analysis with RT-qPCR (TaqMan and SYBR Green assays) confirmed the absence of *Adam19* mRNA expression from exon 6 through 23 in both heart and lung in the KO mice (Fig. S2), additionally validating the absence of exon 11, which encodes the active catalytic site of the ADAM19 protein [19].

Because sufficiently specific ADAM19 antibodies were unavailable, we created the *Adam19-tdTomato* reporter mouse model to visualize the tissue distribution of ADAM19. However, we could not detect fluorescence from the ADAM19-tdTomato fusion protein, possibly due to tdTomato protein misfolding. Nevertheless, we detected tdTomato mRNA transcripts (Fig. S2) and protein (data not shown) in the knockout lung tissue. The Adam19 transcript from the KO contains only the first five exons, representing only 12% (333 nucleotides) of the full-length *Adam19* open reading frame (2760 nucleotides).

### Differential Gene Expression and Gene Set Enrichment Analysis

Differential gene expression analysis revealed few significant changes in gene expression patterns in the lungs of KO compared to WT mice. We observed increased expression of pituitary tumor-transforming gene 1 (*Pttg1*), karyopherin subunit alpha 2 (*Kpna2*), and CD300 molecule like family member g (*Cd300lg*) in KO lungs. (Table 3). Other genes in KO that have increased expression include *Rpl14-ps1*, *Gm21970*, and *Gm11131*. (Table 3). The KO lungs had decreased *AA465934*, *Gm18860*, *Gm12663*, and Gm10184. Consistent with expectation, the WT mice did not exhibit the *Adam19-tdTomato* fusion gene expression.

Gene set enrichment analysis of genes differentially expressed between *Adam19* KO and WT in the lung revealed enrichment for signaling pathways, including MYC_targets_v1, oxidative_phosphorylation, E2F_targets, unfolded_protein_response, protein_secretion, TNFA_signaling_via_NFkB, G2M_checkpoint, DNA_repair, and mitotic_spindle (Table S1).

### Embryo Organogenesis

A pathologist evaluated three E18.5 KO embryos and two E18.5 WT littermates for tissue or organ abnormalities and did not identify lesions associated with tetralogy of Fallot in the heart (overriding aorta, pulmonic stenosis, ventricular septal defect, and right ventricular hypertrophy) as previously reported [19, 30] nor abnormalities in the lungs, diaphragms, and adrenal glands (Fig. S3).

### Reduced Body Weight, Shorter Tibial Length, and Altered Body Composition in *Adam19* KO Mice

**Figure 2:**
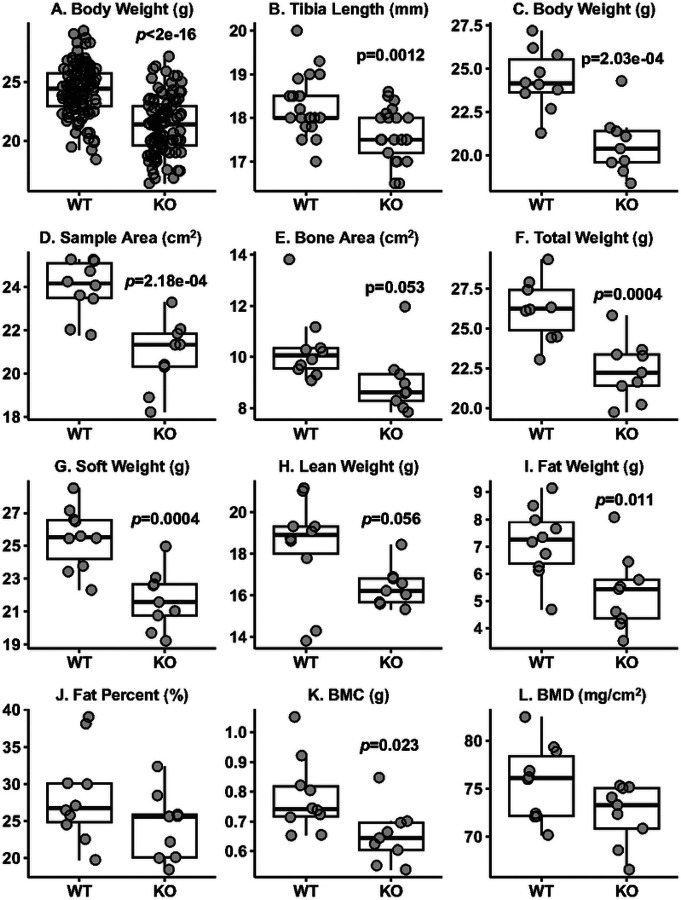
Adam19 KO mice have reduced body weight, shorter tibia length, and altered body composition. A. Body weight measured using a top-loading scale (WT: n=114, KO: n=104). B. Tibia length measured using a ruler (WT: n=24, KO: n=20). C: Body weight of mice for measuring body composition. D-L: Body composition parameters obtained using Dual-energy X-ray Absorptiometry (WT: n=10, KO: n=9). Total weight = soft weight + bone mineral content (BMC), soft weight = lean weight + fat weight, Fat % = fat weight/soft weight in percentage, BMD = bone mineral density = BMC/bone area. p values < 0.05 for differences in the parameter by genotype are displayed (A-L).

We weighed 114 WT and 104 KO mice at 9–13 weeks. Age-adjusted body weight (Fig. 2A) was significantly lower in *Adam19* KO than in WT mice. WT mice continued gaining weight through the assessment period, while the KO stopped gaining weight at nine weeks (Fig. S5). Tibia length was shorter in KO than in WT (20 KO and 24 WT) (Fig. 2B). Using DXA, we measured body composition on 10 KO and 10 WT mice 9.9 to 13.1 weeks and subsequently excluded one outlier KO. In addition to the lower body weight (Fig. 2C), compared to WT, KO had reduced sample area (Fig. 2D), bone area (Fig. 2E), total weight (Fig. 2F), soft weight (Fig. 2G), lean weight (Fig. 2H), fat weight (Fig. 2I), and bone mineral content (BMC) (Fig. 2K). There were no significant differences in %fat (Fig. 2J) or bone mineral density (BMD) (Fig. 2L). Values for body weight and body composition plotted in Figure 2 are shown in Table 1.

### Pulmonary Function Parameters Altered in *Adam19* Deficient Naïve Mice.

**Figure 3:**
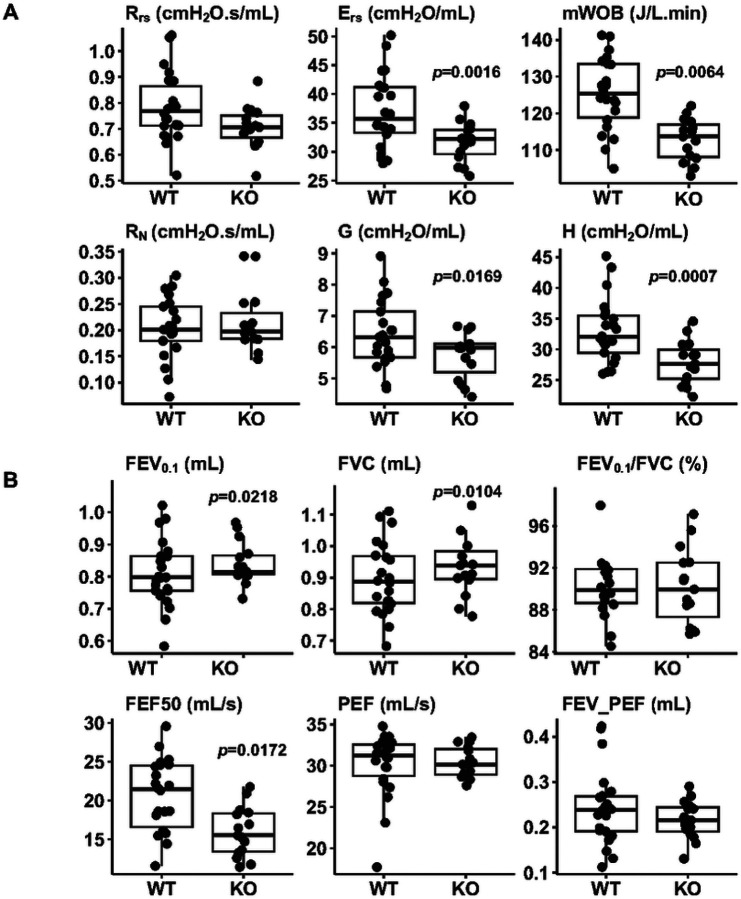
Adam19 deficiency alters (A) baseline mechanics and (B) spirometry parameters determined by flexiVent. n=22 for WT, n=15 for KO. R_rs_=resistance of the respiratory system; E_rs_=elastance of the respiratory system; mWOB=minute work of breathing (the work required to breath-in on a minute basis); J=joule (one joule is the work required to move 1 liter of gas through a 10-cmH_2_O pressure gradient). R_N_=Newtonian resistance; G=tissue damping; H=tissue elastance; FEV_0.1_=forced expiratory volume in 0.1 s; FVC=forced vital capacity; FEV_0.1_/FVC = the ratio of FEV_0.1_ over FVC in %; FEF50=Forced expiratory flow at 50% FVC; PEF=Peak expiratory flow; FEV_PEF=Forced expiratory volume at peak expiratory flow. p<0.05 for differences in the parameter by genotype are displayed.

We measured baseline lung function in 37 mice (22 WT and 15 KO). *Adam19* KO mice exhibited reduced elastance of the respiratory system (E_rs_), minute work of breathing (mWOB), tissue damping (G), and tissue elastance (H) (Fig. 3A). Additionally, forced expired flow at 50% FVC (FEF50) was lower in the KO compared to WT mice (Fig. 3B). However, KO had higher FEV_0.1_ and FVC (Fig. 3B). No genotype differences were observed in resistance of the respiratory system (R_rs_) or Newtonian resistance (R_N_) (Fig. 3A) nor FEV_0.1_/FVC, PEF or FEV_ PEF (Fig. 3B). Additionally, airway responsiveness to methacholine did not differ by genotype (Fig. S6). The means and standard deviations of lung function parameters are shown in Table 2.

### Airway responsiveness to methacholine attenuated in *Adam19* Deficient Mice exposed to LPS.

**Figure 4:**
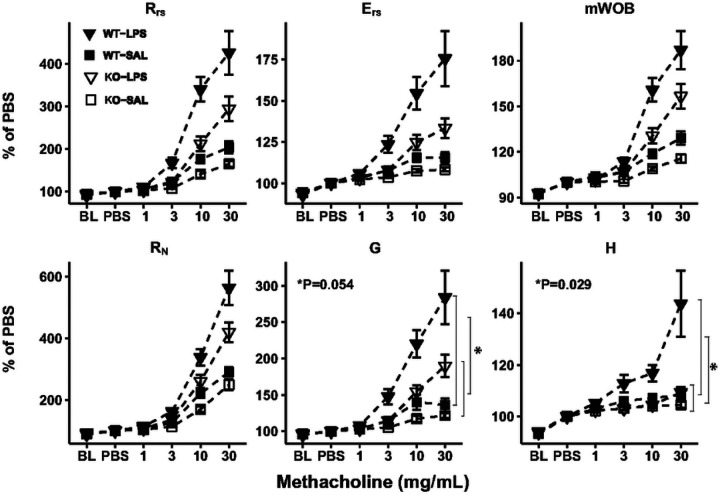
*Adam19* deficient mice have reduced airway responsiveness to methacholine following LPS exposure. The maximum response to methacholine at each dose was expressed as a percentage of the maximum response at PBS. Means and standard errors of means are indicated as bar lines. R_rs_=resistance of the respiratory system; E_rs_=elastance of the respiratory system; mWOB=minute work of breathing; R_N_=Newtonian resistance; G=tissue damping; H=tissue elastance; BL=baseline; PBS=phosphate buffered saline. *p values ≤ 0.05 are shown for the genotype difference of the slope difference of the response to methacholine following LPS exposure (vs. saline). n=13 for WT-Saline, n=20 for WT-LPS, n=14 for KO-Saline, n=21 for KO-LPS

*Adam19* has been shown to promote inflammation in prior literature [17, 18, 31]. Therefore, we assessed genotype differences in airway responsiveness induced by LPS. We did not detect differences in LPS effects (vs. saline) across genotypes for baseline lung function parameters (Fig. S7). However, LPS-induced differences (vs. saline) in the slope estimates for the methacholine dose-response curves were lower in KO than WT for tissue damping (G) and tissue elastance (H) (Fig. 4).

### Immune Cell Differential Analysis in BALF in Mice Following LPS Exposure

**Figure 5:**
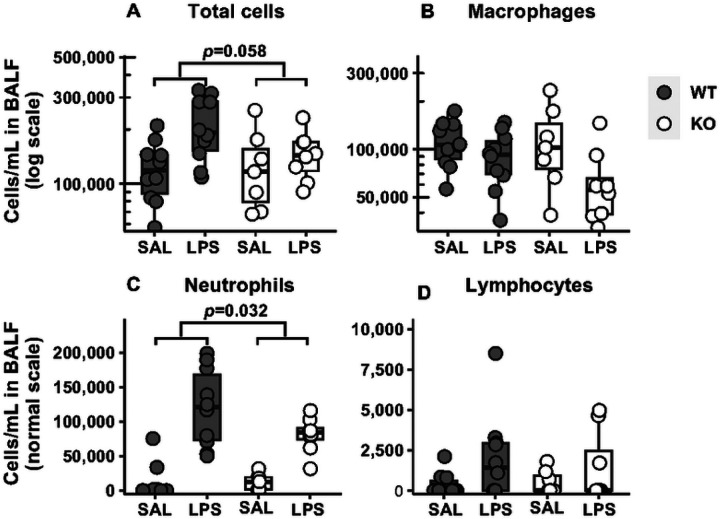
Analysis of differential immune cell counts in bronchoalveolar lavage fluid (BALF) in mice following LPS exposure. Increases in neutrophil number (C) induced by LPS (vs. saline) were lower in Adam19 KO than in WT mice (46% fewer cells, p=0.032). The degree of increase in total cells (A) following LPS (vs. saline) was lower in KO (the ratio of the LPS effect between the KO and WT = 0.61, 95% confidence interval (CI) = 0.36–1.02, p=0.058). No genotype differences of LPS effects were identified for macrophages (B) and lymphocytes (D). SAL=saline, WT: n=10 (SAL), 10 (LPS); KO: n=7 (SAL), 8 (LPS).

As expected, counts of total cells and neutrophils increased in WT and KO mice following LPS exposure. However, the degree of increase in neutrophil counts was lower in KO than in WT (46% fewer cells, *p*=0.032, Fig. 5C). Similarly, the degree of increase in total cells was lower in the KO compared to WT (Fig. 5A). These results suggest that the *Adam19* KO mice show reduced responsiveness to LPS compared to WT regarding immune cell profiles. There were no genotype differences in LPS-induced changes in macrophage or lymphocyte counts (Fig. 5B, 5D).

### Cytokine Analysis in BALF in Naïve and LPS Exposed Mice

As expected, we observed increases in several inflammatory cytokines in response to LPS vs. saline (IL-6, KC, MCP-1, MIP-1a, MIP-1b, and TNF-a); these did not differ by genotype (Fig. S8). IL-1b and IL-2 levels in all mice were below the detection range (not shown).

## Discussion

*Adam19* has been consistently associated with pulmonary function in human GWAS. However, GWAS alone cannot establish causality. Mouse models are useful in investigating the causal role of loci identified in GWAS in pulmonary function. We successfully generated a novel *Adam19* knockout mouse model and confirmed gene disruption through RNA-Seq and RT-qPCR analysis. Contrary to previous publications, our KO mice are viable and generally healthy, without the lethal cardiac abnormalities reported in earlier studies [19, 30].

We considered factors potentially contributing to the discrepancy in the viability of our KO compared to prior work [19, 30]. Firstly, methods for producing the knockout differed between studies. Kurohara *et al*. [19] replaced exons 10 through 12 with an antisense Neomycin resistance cassette, and Zhou et al. [30] introduced a gene trap 3’ of exon 14. We replaced exons 6 and 7 with an in-frame tdTomato construct.

Second, genetic disruption of a multi-exon locus, like *Adam19*, may generate novel transcript variants through alternative splicing, some of which may result in a neomorph that rescues ADAM19 deficiency. In contrast, others may be potentially toxic in the absence of ADAM19. Our RNA-Seq analysis was designed to detect alternative splice variants but identified no alternative *Adam19* splice variants and no active transcription from exons 8 through 23. Interestingly, we detected a novel splice variant in the lung, splicing from exon 5 to exon 8. However, this transcript led to a near-immediate nonsense mutation. Thirdly, the remaining gene structure in a knockout might influence its interaction with other genes or proteins, leading to different functional consequences. Our knockout mice expressed only the first five exons of *Adam19*, which do not include the sequences that encode the active catalytic sites of ADAM19. Transcriptions of exons 8 through 23 were nearly absent, providing confidence that no functional ADAM19 metalloproteinase domains were formed. While both genetic constructs in previous studies [19, 30] disrupt metalloprotease function, they may have generated truncated ADAM19 proteins that could interact with other proteins in a non-productive or dominant-negative manner.

Additional explanations for differences in the viability of the KO across studies include differences in the genetic background of the mouse lines used. Our *Adam19*-deficient allele was generated in 129S ES cells and subsequently maintained on the 129S6/SvEvTac background, whereas the other studies used mice with a mixed genetic background of 129Sv and C57BL6/J [19, 30]. Further, in the prior work, there was variability in the penetrance of the observed cardiac phenotypes. In Kurohara’s ADAM19-deficient line, some mice survived to adulthood without severe cardiac defects besides enlarged hearts [19]. Partial penetrance of lethal phenotypes is common, so this phenotype variability is not surprising. However, it does suggest that the genetic disruption of *Adam19* is more complex than initially envisioned during our gene targeting design.

We do not know why our *Adam19*-deficient mice were viable without the noticeable cardiac defects observed in prior work [19, 30]. However, all available evidence and data strongly indicate that we had a functional knockout of the canonical ADAM19 protein. Our knockout mice unlikely retained ADAM19 activity, given that exons 1 to 5 only encode for the first 111 of 920 amino acids of canonical ADAM19 protein but none of the active sites of metalloproteinase domains. In addition, *Adam19*’s first five exons appear to have higher expression in heart and lung samples in KO than wildtype, possibly caused by either a feedback mechanism attempting to compensate for functional loss of *Adam19* or by the absence of appropriate 3’ UTR elements for the consistent transcript turnover in mutant samples or both.

Our *Adam19* KO animals exhibited several notable phenotypic differences compared to their WT littermates, including reduced body weight, decreased tibia length, and altered body composition. Inoue et al. reported that *Adam19* was involved in osteoblast differentiation in mice [32], which may help explain why our *Adam19* knockouts have shorter tibias. Weerasekera et al. demonstrated a correlation between high ADAM19 expression in human peripheral blood mononuclear cells and BMI, relative fat, and TNF levels [31]. They also observed increased Adam19 mRNA and ADAM19 protein in the liver tissue of mice fed a high-fat diet (HFD). In contrast, neutralizing ADAM19 protein with its antibody resulted in weight loss, reduced white fat accumulation, and decreased TNF protein levels in the liver of HFD-fed mice. These published findings provide insights into our observations of smaller body sizes, reduced body weight, and altered body compositions. In addition, our differential gene expression and GSEA results revealed some supporting clues. *Kpna2* is associated with body weight and BMI in human GWAS [33]. We observed decreased *Kpna2* expression in our KO. Decreased *Cd300lg* expression is associated with increased intramyocellular lipid content and reduced fasting forearm glucose uptake by regulating metabolism in humans [34]. Our knockout mice had increased *Cd300lg* expression, indicating its role in reducing body weight and composition. Our GSEA exhibited the enrichment of differentially expressed genes in multiple pathways related to cell proliferation and metabolism, which could partially explain the anthropometric phenotype in our KO. Collectively, our data support the role of ADAM19 in regulating growth and body weight development.

From human GWAS, it is impossible to predict the direction of effect that knocking out *Adam19* would have on lung function in mice. Human GWAS have identified hundreds of single nucleotide polymorphisms (SNPs) in or near *ADAM19*, significantly associated with lung function [2, 3, 5, 7, 9, 10, 35–37]. Although the minor alleles of sentinel SNPs have been associated with lower FEV_1_/FVC and FEV_1_ and increased risk of COPD, the minor allele of other genome-wide significant variants was associated with higher FEV_1_/FVC and FEV_1_ [2, 3, 5, 7, 9, 10, 35–37]. Hundreds of ADAM19 variants also implicate significant expression quantitative trait loci in lung tissue from Genotype-Tissue Expression (GTEx), with associations including increased and decreased expression depending on the variant (the GTEx Portal: https://gtexportal.org/home/ was accessed 30 September 2023). Given the large number of *ADAM19* variants associated with lung function, gene expression, and the range of effects for individual SNPs, it is impossible to identify a single causal variant or the overall direction of effect across associated variants. This is a known limitation of GWAS [38], highlighting the importance of follow-up research using mouse models.

Critical to comparison with human GWAS, the *Adam19* KO mice also displayed altered baseline pulmonary function parameters, namely decreased elastance of the respiratory system, minute of work of breathing, tissue damping, tissue elastance, and declined forced expiratory flow at 50% forced vital capacity, as well as increased FEV_0.1_ and FVC. Because our KO had a lower body weight, which may affect lung function, we adjusted all statistical analyses of lung function parameters for weight. Our data provide compelling evidence for the causal role of ADAM19 in pulmonary function, confirming the findings observed in human GWAS. However, the precise molecular mechanisms underlying these observations remain unknown. ADAM19 cleaves NEUREGULIN-1 (NRG1), an erythroblastic leukemia viral oncogene homolog (ERBB) receptor tyrosine kinases ligand. ERBB receptor ligands NRG1 and epidermal growth factor affect fetal surfactant synthesis in the developing mouse lungs [39].

Moreover, ADAM19 has been implicated in non-proteolytic functions, such as regulating neuromuscular junctions in murine embryos through Eph family receptor-interacting proteins (EPHRIN)-A5/EPHRIN-A4 signaling [40]. In addition, the cytoplasmic tail of ADAM19 has several Src homology 3 (SH3) binding sites that regulate protein-protein interactions. ADAM19 binds strongly to the scaffolding protein tyrosine kinase substrate with five SH3 domains and the Src tyrosine kinase, potentially influencing cytoskeletal functions that impact cell motility, contractility or tissue development [41]. Therefore, disruption of ADAM19 may have important effects on lung development, neuromuscular functions, tissue elastance, contractility, or other unidentified signaling processes.

Our differential gene expression findings also lead to the direction of genes related to lung physiology and pathology. For example, increased *Kpna2* expression may contribute to altered lung function, consistent with publications that *KPNA2* genetic variation is associated with FEV_1_/FVC in human GWAS [2] and plays a role in lung cancer [42]. Our KO had increased Pttg1 expression. *Pttg1* is involved in cell cycle regulation [43] and the development of lung cancer [44], suggesting its role in the lungs.

Given our observation of reduced neutrophil infiltration in BALF following LPS exposure in the *Adam19* knockout, we investigated whether airway responsiveness to methacholine differs between KO and WT mice following LPS administration. Notably, our knockout mice showed decreased tissue damping and tissue elastance response to methacholine following LPS exposure compared to WT, indicating an attenuated response to inflammation. ADAM19 facilitates the release of TNF from the cell membrane, promoting an inflammatory response and contributing to the development of inflammatory diseases [17, 18, 31, 45]. Our GSEA identified the enrichment of downregulated differentially expressed genes in TNF signaling pathways in our Adam19 KO mice. This is consistent with these previous findings [17, 18, 31, 45] and helps explain the reduced lung functional response to the inflammation in our knockout mice.

In summary, we created a viable whole-body *Adam19* knockout and used this model to examine the role of *Adam19* in lung function, following up on findings from human GWAS implicating this gene. In addition to smaller body size, the lack of functional *Adam19* resulted in reduced respiratory system elastance, minute work of breathing, tissue elastance, forced expiratory flow at 50% FVC, and increased FEV_0.1_ and FVC. These data provide evidence to support a causal role for *Adam19* in regulating pulmonary function development. Pathway analysis of genes differentially expressed after disruption of Adam19 implicates pathways crucial in lung inflammation, including TNF signaling pathways. Although our study is limited to a descriptive scope and a definitive understanding of mechanisms underlying our findings requires further investigation, our novel *Adam19* KO murine model could be helpful in future studies to dissect the role of this gene in lung function.

## Figures and Tables

**Table 1:** Body composition parameter values by genotype as shown in Fig.2C-L

	WT (n=10)		KO (n=9)		
Parameters	Mean	SD	Mean	SD	*p*-value
**Body Weight (g)**	24.4	1.7	20.6	1.8	2.03e-04
**Sample Area (cm** ^**2**^ **)**	24.0	1.3	20.9	1.6	2.18e-04
**Bone Area (cm** ^**2**^ **)**	10.3	1.4	9.0	1.2	0.053
**Total Weight (g)**	26.3	1.9	22.4	1.9	0.0004
**Soft Weight (g)**	25.5	1.9	21.7	1.8	0.0004
**Lean Weight (g)**	18.3	2.5	16.4	0.9	0.056
**Fat Weight (g)**	7.2	1.3	5.3	1.4	0.011
**Fat Percent (%)**	28.3	6.2	24.3	4.5	0.135
**BMC (g)**	0.8	0.1	0.7	0.1	0.023
**BMD (mg/cm** ^**2**^ **)**	75.6	3.9	72.3	3.1	0.074

SD: standard deviation; BMC: Bone Mineral Content; BMD: Bone Mineral Density = BMC/(Bone Area); Total Weight = Soft Weight + BMC; Soft Weight = Lean Weight + Fat Weight; Fat Percent = (Fat Weight)/(Soft Weight) in percentage. The p-values were based on linear regression adjusting for age.

**Table 2:** Mean and standard deviation of baseline lung function values by genotype as shown in Fig. 3

	WT (n=22)		KO (n=15)		
Parameters	Mean	SD	Mean	SD	*p*-value
**Mechanics**					
R_rs_ (cmH_2_O.s/mL)	0.79	0.13	0.71	0.08	0.0542
E_rs_ (cmH_2_O/mL)	36.98	6.33	31.64	3.37	0.0016
mWOB (J/L.min)	125.36	9.94	112.83	5.77	0.0064
R_n_ (cmH_2_O.s/mL)	0.20	0.06	0.22	0.06	0.8867
G (cmH_2_O/mL)	6.44	1.09	5.73	0.73	0.0169
H (cmH_2_O/mL)	33.13	5.27	27.9	3.52	0.0007
**Spirometry**					
FEV_0.1_ (mL)	0.81	0.10	0.84	0.06	0.0218
FVC (mL)	0.90	0.12	0.93	0.09	0.0104
FEV_0.1_/FVC (%)	90.18	2.77	90.23	3.61	0.4564
FEF50 (mL/s)	20.61	4.63	15.97	3.20	0.0172
PEF (mL/s)	30.10	3.88	30.42	1.85	0.653
FEV_PEF (mL)	0.24	0.08	0.22	0.04	0.7124
**Age and Weight**					
Age (wk)	10.2	0.7	10.5	0.7	0.205
Body Weight (g)	23.3	2.0	21.3	2.5	0.0118

SD-Standard Deviation; R_rs_=resistance of the respiratory system; E_rs_=elastance of the respiratory system; mWOB=minute work of breathing (the work required to breathe in on a minute basis); J=joule (one joule is the work required move 1 liter of gas through a 10-cmH2O pressure gradient); R_N_=Newtonian resistance; G=tissue damping; H=tissue elastance; FEV_0.1_=forced expiratory volume in 0.1 s; FVC=forced vital capacity; FEV_0.1_/FVC = the ratio of FEV_0.1_ over FVC in %; FEF50=Forced expiratory flow at 50% FVC; PEF = Peak expiratory flow; FEV_PEF=Forced expiratory volume at peak expiratory flow.

**Table 3:** Differentially Expressed Genes in Adam19 KO versus WT Mouse Lungs Based on RNA-Seq^a^

Gene Name	Fold Change	Adjusted *p*-value^a^	MGM
Rpl14-ps1	797.9	0.0247	10.64
Gm21970	396.4	0.0162	9.63
Gm11131	33.0	0.0138	6.04
Pttg1	16.3	0.0006	9.32
Kpna2	8.8	0.0291	9.10
Cd300lg	5.4	0.0474	7.47
AA465934	−15.1	0.0044	6.59
Gm18860	−32.1	0.0474	6.01
Gm12663	−55.2	0.0007	6.53
Gm10184	−426.8	0.0138	9.74

MGM means maximum group mean (the highest normalized group mean abundance for each gene)

a Considered statistically significant if false discover rate adjusted *p*<0.05, the absolute value of fold change ≥h1.5 (positive fold change means increase and negative fold change means decrease), and MGM ≥G6. The table was sorted by fold change descending; n=3 per genotype.
